# In silico study to explore the mechanism of *Toxoplasma-*induced inflammation and target therapy based on sero and salivary *Toxoplasma*

**DOI:** 10.1038/s41598-024-63735-z

**Published:** 2024-06-13

**Authors:** Faika Hassanein, Hewida H. Fadel, Amany I. Shehata, Noha Alaa Hamdy, Inas M. Masoud

**Affiliations:** 1 Department of Microbiology & Immunology, Faculty of Dentistry, Pharos University in Alexandria, Alexandria, Egypt; 2 Department of Medical Laboratory Technology, Faculty of Applied Health Sciences Technology, Pharos University in Alexandria, Alexandria, Egypt; 3 Department of Tropical Health, High Institute of Public Health, Alexandria University, Alexandria, Egypt; 4 Department of Clinical Pharmacy & Pharmacy Practice, Faculty of Pharmacy, Alexandria University, Alexandria, Egypt; 5 Department of Pharmacology and Therapeutics, Faculty of Pharmacy, Pharos University in Alexandria, Alexandria, Egypt

**Keywords:** Salivary, Sera, Discrimination, Docking, Drug, Data, Bank, Computational biology and bioinformatics, Drug discovery, Immunology, Microbiology, Biomarkers, Medical research, Risk factors

## Abstract

We aimed to assess salivary and seroprevalence of *Toxoplasma* immunoglobulins in risky populations and evaluate drug docking targeting TgERP. A cross-sectional study was conducted in Alexandria University hospitals’ outpatient clinics. 192 participants were enrolled from September 2022 to November 2023. Anti-*Toxoplasma* IgG and IgM were determined in serum and saliva by ELISA. An in-Silico study examined TgERP’s protein–protein interactions (PPIs) with pro-inflammatory cytokine receptors, anti-inflammatory cytokine, cell cycle progression regulatory proteins, a proliferation marker, and nuclear envelope integrity-related protein Lamin B1. Our findings revealed that anti-*T. gondii* IgG were detected in serum (66.1%) and saliva (54.7%), with 2.1% of both samples were positive for IgM. Salivary IgG had 75.59% sensitivity, 86.15% specificity, 91.40% PPV, 64.40% NPP, 79.17% accuracy and fair agreement with serum IgG. On the other hand, the sensitivity, specificity, PPV, NPV, and accuracy in detecting salivary IgM were 75.0%, 99.47%, 75.0%, 99.47%, and 98.96%. AUC 0.859 indicates good discriminatory power. Examined synthetic drugs and natural products can target specific amino acids residues of TgERP that lie at the same binding interface with LB1 and Ki67, subsequently, hindering their interaction. Hence, salivary samples can be a promising diagnostic approach. The studied drugs can counteract the pro-inflammatory action of TgERP.

## Introduction

*Toxoplasma gondii* is a cosmopolitan zoonotic protozoan parasite^1^. The global prevalence of latent toxoplasmosis in pregnant women was estimated to be 33.8%^2^. The seropositivity for anti- *Toxoplasma* IgG among diabetic patients from Cairo, Egypt was 46%. A higher rate was revealed among pregnant women (67.5%) in Menoufia^3,4^. Humans can be infected by ingesting tissue cysts in uncooked meat or from an infected mother to her fetus. The latter may result in stillbirth, abortion, and congenital abnormalities including chorioretinitis, microcephalus, intracerebral calcifications, and hydrocephalus^5,6^. In addition, infection can be acquired by ingesting oocysts in unsanitary environments via contaminated food and water^7^. While it is asymptomatic and self-limited in immunocompetent individuals, it can cause ocular or neurological disorders in immunocompromised patients^8^.

It can be accurately diagnosed by various methods, including serological tests, polymerase chain reactions, histological examination, and organism isolation^9^. Traditionally, blood samples have been the primary source of serological testing. However, researchers have been exploring alternative sample sources to improve testing convenience and patient compliance^10^. Body fluids, especially blood, saliva, and urine, are rich resources for potential diagnostic biomarkers for a number of diseases^11^. Mahmoud^12^ demonstrated that urine samples could be easily and readily collected without causing any inconvenience to the study participants. The samples showed high sensitivity to *T. gondii* antigens by reverse latex agglutination test and enzyme-linked immunosorbent assay (ELISA) among patients with acute toxoplasmosis. Additionally, several studies have focused on investigating toxoplasmosis in animals. For instance, Huskinson et al.^13^ detected *Toxoplasma* antigens in urine of acutely infected mice as well as patients with acute toxoplasmic encephalitis. Years later, Hu et al.^14^, detected the *Toxoplasma* B1 gene in urine samples of infected mice using the LAMP method. Recently, Xie et al.^11^, revealed the detection of a total of 88 common DE miRNAs and 120 DE novel piRNAs in both serum and urine samples from infected rabbits. Saliva has gained attention as a non-invasive and readily accessible biofluid that may offer a reliable alternative for *T. gondii* serological testing^15^.

Tests using oral fluid have been developed for viral infections including HIV^16^, hepatitis^17^, norovirus^18^, and cytomegalovirus^19^, as well as bacterial infections such as *Helicobacter pylori*^18^, and parasitic infections like *T. gondii* and *Cryptosporidium parvum*^18,20^. Oral fluid comprises crevicular fluid that is notably rich in IgA, IgG, and IgM^21^. Most IgG in saliva is derived from serum and enters the oral cavity via crevicular fluid whereas most IgA in oral fluid is produced in the salivary glands, reflecting both mucosal and systemic immunity^22^. IgM is present at lower concentrations in salivary fluid than IgG and IgA^23^.

The additional complexity of toxoplasmosis is attributed to its tightly regulated life cycle, which alternates between sexual developmental stages in felines and multiple asexual forms in a wide range of intermediate hosts. The host immune response stimulates the tachyzoites to switch back to bradyzoites that encyst in skeletal muscle and brain tissues to establish a chronic infection. All this paid attention to exploring high-efficacy therapy targeting *Toxoplasma* proteins-induced immunological disorders based on serological tests^24^.

Epidemiological studies that could lead to the development of strategies to reduce infection in humans and food animals are made difficult by the lack of tests that can distinguish between the infection routes of ingestion of tissue cysts in undercooked meat and oocysts in cats’ feces^25^. Among the numerous immunogenic proteins in *T. gondii* late embryogenesis abundant domain-containing proteins are crucial for pathogen survival and resistance to high salinity, drought, and freezing conditions. Of these, embryogenesis-related protein “ERP” serves as a sporozoite-specific antigen “*T. gondii* embryogenesis-related protein” (TgERP) and corresponds to the TGME49_076850 gene^25,26^. IgG antibodies against TgERP are more prevalent than non-specific IgG and aid in discriminating between oocyst and bradyzoite infections^27^. While TgERP helps *T. gondii* sporozoites to persist for long periods in harsh environmental reservoirs without a host, its short-lasting nature (about 6–8 months) and the relatively short half-life of anti-TgERP antibodies limit its applications in epidemiological assessment studies^26,28^. The presence of this antibody differentiates infection in pigs caused by sporulated oocysts from tissue cysts and clearly identifies people infected through ingestion of oocysts^26,28^. These factors underscore the importance of exploring the mechanism by which TgERP induces inflammation in infected individuals and may lead to identification of new therapeutic targets.

Many studies have evaluated the role of neutrophils in controlling *T. gondii* infection in mice by enhancing reactive oxygen species and neutrophil extracellular trap (NET) formation. However, few studies have investigated this role in infected humans*.* LB1, a nuclear lamina protein, constitutes a major structural component of the nucleus and regulates many nuclear functions. It is essential for the structural integration of the nuclear envelope, while nuclear envelope rupture and chromatin externalization are hallmarks of NET formation^29^. Down-regulation of LB1 may cause cell senescence and slower cell proliferation^30^.

Additionally, *T. gondii* enhances the recruitment of autologous CD4 + T cells and the production of many cytokines including interferon-gamma (IFN-γ) tumor necrosis factor, interleukin 6 (IL-6), IL-17, and IL-10 by peripheral blood mononuclear cells. Regarding the impact of *T. gondii* on the cell cycle of the host, a recent study revealed that infection with *T. gondii* induces the gene expression of cell cycle protein ki-67, which promotes cell cycle progression and carcinogenesis^31^.

Recently, bioinformatics has become an essential tool in analyzing, explaining, and interpreting disease mechanisms in biomedical studies, aiding in the exploration of therapies^32^. Among these tools, molecular docking and drug docking are the most important to analyze PPI and exploring novel therapies. Milne et al.^33^, revealed that prolonged exposure to *T. gondii* is associated with high prevalence of anti-TgERP IgG antibodies, so we utilized molecular and drug docking techniques to investigate the mechanisms underlying *T. gondii*-related inflammation. Furthermore, we delved into drug docking strategies aimed at targeting pro-inflammatory proteins and cytokines.

Due to the scarcity of studies, our aim was to assess anti-*T. gondii* IgG and IgM in serum and saliva samples of patients recruited to the outpatient clinics of Alexandria University hospitals who are at risk for toxoplasmosis, as well as explore novel therapies targeting key mediators in pathway of *T. gondii*-induced inflammation employing bioinformatics tools including molecular and drug docking, with a focus on targeting TgERP.

## Subjects and methods

### Study design and population

A cross-sectional study was conducted in the outpatient clinics of different Alexandria University hospitals, in Egypt. A total of 192 individuals (84 males and 108 females) with a mean age of 26.3 ± 1.17, were enrolled in the study from September 2022 to November 2023. Informed consent was obtained from all participants or their legal guardians after explaining the study’s purpose and before collecting demographic data, blood, and saliva samples. Immunocompromised individuals enrolled in the study were suffering from rheumatic arthritis, leukemia, cancer, renal disorders, liver cirrhosis, hemodialysis, and chemotherapy, and those of immunosuppressive therapies.

The sample size was calculated using Fisher’s formula of sample size calculation based on prevalence data from a previous study:

$$ {\text{n}} = {\text{z}}^{{2}} {\text{p}}\left( {{1}00 - {\text{p}}} \right)/{\text{d}}^{{2}} , $$

whereby n is the sample size, z is the statistic corresponding to the decided level of confidence, in this case, 95% with a z value of 1.96, p is the prevalence obtained from the previous study, d is the amount of error that can be tolerated by the study, in this case, 5%.

### Data and sample collection

A structured, predesigned questionnaire based on known risk factors was developed. A pilot study was conducted in August 2022 to evaluate the validity and feasibility of the questionnaire. Participants in the current study were interviewed to collect sociodemographic data, personal habits and hygiene information, clinical symptoms, and the presence of chronic diseases.

Blood and saliva samples were collected from every participant. Blood (3 ml) was collected by venipuncture into pre-labeled plain tubes and left to clot at room temperature. Serum samples were separated by centrifugation for 10 min at 3000 rpm. Participants were allowed to chew gum for collecting saliva samples (3 ml) using sterile pasture pipettes^34^, in 15 ml falcon tubes containing saline borate buffer (NaCl 0.1 M, Borate buffer 0.05 M, pH 7.2) kept in an ice box and centrifuged at 3000×*g* for 15 min. at 4 °C. The supernatant was collected, an equal volume of absolute ethanol was added, vortexed and stored at − 12 °C by at least one hour. Then centrifuged at 4 °C at 10,000 × *g* for 5 min, the supernatant was discarded, and the pellet was re-suspended in 0.2 mL of saline borate buffer that to increase the concentrations of IgG and IgM in the saliva^20^. Both sera and saliva samples were aliquoted, labeled, and stored at – 20 °C until serological investigations were performed by ELISA kits to detect IgG and IgM.

### Conventional serological investigations

All serum and saliva samples were thawed to room temperature and tested for anti- *Toxoplasma* IgG and IgM using commercial ELISA kits (Diapro Elisa Kit, Italy) following the manufacturer’s instructions. Positive and negative controls were included to ensure the integrity of reagents and technical procedures, and results were interpreted according to instructions provided with the reagent kits^35,36^.

### Statistical analysis

Data were analyzed using IBM SPSS statistics, version 25.0 (IBM Corp., Armonk, NY, USA). Categorical variables were assessed using the Chi-square test or Fisher’s exact test, whichever is suitable. Associations and differences were considered statistically significant at P < 0.05. Sensitivity, specificity, positive and predictive values (PPV and NPV), the receiver operating characteristic (ROC) curve analysis were also conducted. The ROC curve data provided the accuracy of each test with area under the curve (AUC) measurements and Cohen’s Kappa coefficient (K). The Cut-off was calculated according to the Youden index.

### In silico study

Initially we conducted molecular docking firstly to examine the mechanism of action of TgERP, followed by drug docking to explore drugs with high affinity towards TgERP. Specifically, those which can interfere with the binding of TgERP with lamin B1 and Ki67, thereby potentially mitigating the dysregulatory effect of TgERP on nuclear proteins. Surprisingly, our findings revealed that the studied drugs targeted the epitope of TgERP that exert antigenicity to B cell, thus, these drugs may counteract the pro-inflammatory action of TgERP”.

### Molecular docking

To achieve our objective, an in-Silico study was carried out to analyze the PPI between TgERP and pro-inflammatory cytokines receptors including IL-6R, IL-17R, the anti-inflammatory cytokine (IL-10), cell cycle progression regulatory proteins such as Ki67 which serves as a proliferation marker and a nuclear envelope integrity-related protein LB1.

Molecular docking was performed to explore the PPI between TgERP and several human proteins involved in inflammation such as IL-6R (PDB:1N26), IL-17R (PDB:5N9B), IL-10 (PDB:2H24) as well as proteins related to cell cycle progression, such as Ki67 (PDB:5J28) and LB1 (pdb:3UMN) as a possible mechanism for inducing inflammation in patients infected with *Toxoplasma*. The molecular docking of proteins was conducted using Cluspro, a clustering program for evaluating PPI, and Pymol was utilized for visualizing PPI^37^. To evaluate the accuracy of docking, root mean standard deviation was conducted via TM-align^38^. Additionally, binding affinity (∆G) in Kcal/mol and dissociation constant (Kd) values were extracted by Prodigy^39^.

### Drug docking

We used ChemSktech for drugs drawing, Avogadro energy for drug optimization, Spdby for energy optimization, Discovery Studio 2021 as a visualization tool of ligand-receptor interactions, and iGdemodock for drug docking^40^. We employed predictive modeling to determine the B-cell epitope of TgERP^41^. Also, predictions for MHCI binding were conducted to identify T-cell epitopes using the IEDB analysis resource NetMHCpan (ver. 4.1) tool^42^.

### Ethics information

The study was approved by the ethics committee of the Faculty of Medicine, Alexandria University (0305694). All methods were performed following the Declaration of Helsinki.

### Consent to participate

The manuscript has been read and approved by all authors

## Results and discussion

### Serological analysis

Table 1 shows that over half of the participants were females, under 30 years old, reside urban area, consuming untreated water and uncooked processed meat, and eating food outdoors. However, approximately 66% reported washing hands before eating and not owning any pets.

**Table 1:** Distribution of the studied sample according to some Socio-demographic, environmental characteristics, and personal hygiene.

Characteristics	N	%
Gender		
Males	84	43.8
Females	108	56.2
Age in years		
< 30	107	55.7
≥ 30	85	44.3
Educational level		
Illiterate and read & write	31	16.2
Preuniversity	119	62.0
Medium	35	18.2
University	7	3.6
Area of resident		
Urban	107	55.7
Rural	85	44.3
Job		
Not work	123	64.1
Work	69	35.9
Groups participated		
Immunocompetent	96	50.0
Immunocompromised	96	50.0
Source of drinking water		
Filter	89	46.4
*Untreated	103	53.6
Washing hands before eating		
No	64	33.3
Yes	128	66.7
Eating foods outdoor		
No	91	47.4
Yes	101	52.6
Eating undercooked processed meat		
No	72	37.5
Yes	120	62.5
Contact with cats/animals		
No	125	65.1
Yes	67	34.9

*Tap in, tap out, pump, zeir.

A high *T. gondii* IgG rates was detected in both sera and salivary samples examined (66.1% and 54.7%, respectively), with a statistically significant difference (^McN^p = 0.001). Conversely, IgM showed equal lower rates (2.1%) as depicted in Table 2. That may be attributed to inadequate hygiene practices, consumption of outdoors food, undercooked processed meat, and untreated water, and contact with cats and animals. Earlier studies have supported our findings, reporting *T. gondii* infections rates among mothers during the second or third trimester as high as 68%^43^. Macre et al.^44^ revealed a 50% salivary anti-*T. gondii* IgG among students. Although the seroprevalence in our study was high, it was relatively lower compared to a recent study in Lyon (France), which reported serum *T. gondii* IgG and IgM rates of 100% and 94.12, respectively, and salivary IgG and IgM rates of 94.9% and 100%, respectively by Li et al.^10^. Robert-Gangneux et al.^45^ revealed that toxoplasmosis seroprevalence of 100%, 90%, and 89%, among patients with cerebral, pulmonary, and post-transplant toxoplasmosis, respectively. However, lower rates of IgG and IgM in serum were observed among pregnant women (57.3%, and 3.5%, respectively)^46^. In Brazil, an endemic setting for toxoplasmosis, sera IgG, IgM, and salivary IgA were 49.8%, 5.7%, and 31.65, respectively^47^. Nayeri et al.^15^, reported salivary IgG, IgM, and IgA rates of 18.66%, 15%, and 49.18%, respectively, in Iran. In Egypt, IgG seropositivity rates were 46% and 45% among diabetic patients according to studies by Hemida et al.^48^ and Khattab et al.^49^, respectively. Pregnant women in Nigeria exhibited low rates of IgG and IgM (26.6% and 5.7%, respectively)^50^. Among diabetic patients and patients enrolled from hospitals in five provinces of China, IgG rates were 28.6% and 5.13%, respectively^51,52^. Finally, 20% and 25% of the saliva of HIV patients were positive for IgG and IgM respectively^53^. These studies attributed the low rates of toxoplasmosis to several factors, including increased attention to health and hygiene, consumption of fully cooked food and boiled water, thorough washing raw vegetables, and avoidance of contact with cats and animals, beside economic development and improvements in quality of life.

**Table 2:** Comparison between ELISA techniques in detecting serum and saliva IgG and IgM.

Conventional serological investigations of salivary and serum immunoglobulins				
Immunoglobulins analysis	Serum (n = 192)	Saliva (n = 192)	Test of sig	p
Qualitative analysis				
IgG	127 (66.1%)	105 (54.7%)	χ^2^ = 66.146*	^McN^p = 0.001*
IgM	4 (2.1%)	4 (2.1%)	χ^2^ = 106.474	^McN^p = 1.000
Quantitative analysis				
IgG				
Min.–Max	0.07–21.80	0.20–17.55	Z = 0.806	0.420
Mean ± SD	6.43 ± 5.06	5.75 ± 4.96		
IgM				
Min.–Max	0.10–2.10	0.10–2.30	Z = 7.855*	< 0.001*
Mean ± SD	0.28 ± 0.33	0.16 ± 0.28		

*SD* standard deviation, *McN* McNemar test, *Z* Wilcoxon signed ranks test.

p: p value for comparing between Serum and Saliva.

*Statistically significant at p ≤ 0.05.

#### Risk factors associated with toxoplasmosis among the samples examined

Our findings indicate that immunocompromised individuals are at a higher risk of toxoplasmosis infection compared to immunocompetent individuals, as evidenced by respective risk factors of 2.469-fold and 7.857-fold of infection in serum and saliva samples (Table 3). This finding is consistent with Wang and his colleagues, who demonstrated a 2.89-fold higher odds of infection immunocompromised individuals, and recommended appropriate prevention and control measures for these susceptible populations^54^.

**Table 3:** *Toxoplasma gondii* immunoglobulins among the studied sample according to sociodemographic data.

Parameters	Total examined	IgG						IgM					
		Serum (n = 127)	*p*_*1*_	*OR*_1_ (95% C.I.)	Saliva (n = 105)	*p*_*2*_	*OR*_2_ (95% C.I.)	Serum (n = 4)	*p*_*1*_	*OR*_1_ (95% C.I.)	Saliva (n = 4)	*p*_*2*_	*OR*_2_ (95% C.I.)
Groups													
Immunocompetent	96	54 (56.3)	0.004*	1.000	30 (31.3)	< 0.001*	1.000	0 (0.0)	0.996	1.000	0 (0.0)	0.996	1.000
Immunocompromised	96	73 (76.0)		2.469 (1.330 − 4.581)	75 (78.1)		7.857 (4.108 − 15.026)	4 (4.2)		NA	4 (4.2)		NA
Gender													
Males	84	55 (65.5)	0.863	1.000	41 (48.8)	0.150	1.000	1 (1.2)	0.458	1.000	1 (1.2)	0.458	1.000
Females	108	72(66.7)		1.055 (0.578 − 1.925)	64 (59.3)		1.525 (0.859 − 2.710)	3 (2.8)		2.371 (0.242 − 23.217)	3 (2.8)		2.371 (0.242 − 23.217)
Age (years)													
< 30	107	62 (57.9)	0.008*	1.000	44 (41.1)	< 0.001*	1.000	1 (0.9)	0.244	1.000	2 (1.9)	0.816	1.000
≥ 30	85	65 (76.5)		2.359 (1.255 − 4.435)	61 (71.8)		3.639 (1.979 − 6.693)	3 (3.5)		3.878 (0.396 − 37.970)	2 (2.4)		1.265 (0.174 − 9.172)
Residence													
Urban	107	80 (74.8)	0.005*	2.396 (1.301 − 4.413)	70 (65.4)	0.001*	2.703 (1.502 − 4.864)	3 (2.8)	0.447	2.423 (0.248 − 23.722)	3 (2.8)	0.447	2.423 (0.248 − 23.722)
Rural	85	47 (55.3)		1.000	35 (41.2)		1.000	1 (1.2)		1.000	1 (1.2)		1.000
Jobs													
Not work	123	76 (61.8)	0.090	1.000	58 (47.2)	0.006*	1.000	2 (1.6)	0.559	1.000	2 (1.6)	0.559	1.000
Work	69	51 (73.9)		1.752 (0.916 − 3.353)	47 (68.1)		2.394 (1.291 − 4.441)	2 (2.9)		1.806 (0.249 − 13.114)	2 (2.9)		1.806 (0.249 − 13.114)

OR_1_: Odds ratio for negative serum^®^ and positive serum, *p*_*1*_*:* P-value for positive serum. OR_2_: Odds ratio for negative saliva^®^ and positive saliva, *p*_*2*_*:* P-value for Positive Saliva.

*C.I* confidence interval, *LL* lower limit, *UL* upper limit, *NA* not applicable.

*Statistically significant at p ≤ 0.05.

Although females exhibited higher risks for IgG and IgM in both serum and salivary samples [(1.055-fold), (1.525-fold) for IgG, and (2.371-fold) for IgM] compared to males, yet the difference wasn’t statistically significant (Table 3). That agrees with reports from Egypt in the survey conducted et al.-Hussien University Hospital (7.3% vs. 6.2%) and in the diabetes clinic at the Research Institute of Ophthalmology (70.4% vs. 29.6%)^49,55^.

Furthermore, individuals aged 30 years or older were at significantly higher risk for IgG in both sera and saliva (OR = 2.359 and 3.639 respectively) compared to those younger than 30 years. Regarding IgM, slightly non-significant higher ORs were detected among older individuals compared to younger ones (3.878 and 1.265, respectively) (Table 3). Inconsistent with our findings, several studies showed a significant increase in toxoplasmosis seroprevalence by age in Brazil, Nigeria, Egypt, and China^49,52,56–58^. However, in 2017, Hemida et al.^55^, reported a non-significant negative relation between seropositivity and the increasing age.

Our results demonstrate that urban residents were approximately two times more likely to have IgG and IgM in both serum and saliva than rural residents. The difference was statistically significant only for IgG (Table 3). However, several studies have reported no statistical difference in seroprevalence between rural and urban areas (p-value < 0.05)^49,50,55,59^. In Brazil, Avelar and his colleagues^60^ reported higher seroprevalence among pregnant women residing in rural areas, attributing these results to low socioeconomic levels, difficulties in obtaining health services, increased exposure, and lack of awareness about the mode of transmission.

Moreover, employed individuals showed a higher risk of being IgG salivary and seropositive (2.394 & 1.752 respectively), and IgM salivary and seropositive (1.806-fold) compared to unemployed ones. However, the only significant difference was observed for salivary IgG (Table 3). This may be attributed to eating outdoors food, consumption of undercooked processed meat, and improperly washed raw vegetables. This finding aligns with that reported by Khabisi et al.^61^, who attributed the infection among students to contamination of playgrounds with sporulated oocyst, and among housekeepers to keeping cats and cleaning contaminated vegetables.

Concerning personal hygiene parameters, our findings show that individuals using untreated drinking water exhibited a higher but insignificant risk of being IgG positive in saliva and serum (OR = 1.253 & 1.307 respectively) compared to those using filtered water. Similar trends were observed for IgM in both samples (OR = 2.640), (Table 4). This finding is consistent with reports from various parts of the world, suggesting that the source of drinking water can impact *Toxoplasma* transmission^59^. Recently, Okojokwu et al.^50^ found a higher frequency of infection among those using pipe-borne water as their drinking water source, and the same was reported by Singh et al.^46^.

**Table 4:** *Toxoplasma gondii* immunoglobulins among the studied sample in relation to personal hygiene.

Parameters	Total examined	IgG						IgM					
		Serum (n = 127)	*p*_*1*_	*OR*_1_ (95% C.I.)	Saliva (n = 105)	*p*_*2*_	*OR*_2_ (95% C.I.)	Serum (n = 4)	*p*_*1*_	*OR*_1_ (95% C.I.)	Saliva (n = 4)	*p*_*2*_	*OR*_2_ (95% C.I.)
Source of water drinking													
Filter	89	56 (62.9)	0.381	1.000	46 (51.7)	0.438	1.000	1 (1.1)	0.404	1.000	1 (1.1)	0.404	1.000
Untreated	103	71 (68.9)		1.307 (0.718 − 2.381)	59 (57.3)		1.253 (0.709 − 2.217)	3 (2.9)		2.640 (0.270 − 25.842)	3 (2.9)		2.640 (0.270 − 25.842)
Washing hands before eating													
No	64	50 (78.1)	0.014*	2.365 (1.186 − 4.717)	41 (64.1)	0.066	1.783 (0.962 − 3.304)	3 (4.7)	0.116	6.246 (0.637 − 61.289)	2 (3.1)	0.483	2.032 (0.280 − 14.769)
Yes	128	77 (60.2)		1.000	64 (50.0)		1.000	1 (0.8)		1.000	2 (1.6)		1.000
Eating food outdoor													
No	91	51 (56.0)	0.005*	1.000	42 (46.2)	0.025*	1.000	1 (1.1)	0.384	1.000	2 (2.2)	0.916	1.000
Yes	101	76 (75.2)		2.384 (1.292 − 4.401)	63 (62.4)		1.934 (1.087 − 3.441)	3 (3.0)		2.755 (0.281 − 26.968)	2 (2.0)		0.899 (0.124 − 6.516)
Eating undercooked processed meat													
No	72	39 (54.2)	0.007*	1.000	29 (40.3)	0.002*	1.000	1 (1.4)	0.607	1.000	1 (1.4)	0.607	1.000
Yes	120	88 (73.3)		2.327 (1.258 − 4.305)	76 (63.3)		2.561 (1.406 − 4.665)	3 (2.5)		1.821 (0.186 − 17.840)	3 (2.5)		1.821 (0.186 − 17.840)
Contact with cats and animals													
No	122	72 (57.6)	0.001*	1.000	56 (44.8)	< 0.001*	1.000	1 (0.8)	0.131	1.000	2 (1.6)	0.528	1.000
Yes	70	55 (82.1)		3.374 (1.645 − 6.919)	49 (73.1)		3.354 (1.760 − 6.393)	3 (4.5)		5.812 (0.593 − 57.008)	2 (3.0)		1.892 (0.261 − 13.745)

OR_1_: Odds ratio for negative serum^®^ and positive serum, *p*_*1*_*:* P-value for positive Serum. OR_2_: Odds ratio for negative saliva^®^ and positive Saliva, *p*_*2*_*:* P-value for positive Saliva.

*C.I* confidence interval, *LL* lower limit, *UL* upper Limit. *Statistically significant at p ≤ 0.05.

Additionally, a significant risk factor for sera IgG was detected among those who didn’t wash their hands before eating (OR = 2.365). Meanwhile, those who ate food outdoors, ate undercooked processed meat, and had a contact with cats or other animals exhibited a statistically significant higher risk of IgG in both sera and salivary fluids [OR = 2.384 & 1.934), (2.327 & 2.561), and (3.374 & 3.354-fold), respectively], (Table 4). Consistent with our findings, Friesema et al.^62^, reported that the occasional or never washing hands before food preparation was found to be a risk factor (OR = 4.1) for infection. In 2017, Iddawela et al.^63^, reported that housewives can accidentally become infected by ingesting tissue cysts or tachyzoites from animal blood during meat handling, such as in cutting and washing meat before cooking. Moreover, several studies identified a significant association between *T. gondii* seropositivity and the consumption of raw and undercooked meat^64–66^. Our findings were aligning with those of Abu^67^, who reported that cats play a key role in the epidemiology of *T. gondii*, being the sole source of infective oocyst contamination in the environment. Additionally, the study conducted by Ibrahim et al.^68^ reported that a significantly high percentage of pregnant women who had contact with cats (16.36%) and who consumed undercooked mutton (57.37%) tested positive for *T. gondii*. These findings contradict studies from Nigeria and Egypt, that reported that cat ownership was not associated with *T. gondii* seropositivity^50,69,70^. Furthermore, in 2019, it was reported that there was a statistically significant difference in seroprevalence concerning the consumption of undercooked meat, contact with cats and contact with soil (85.2%). This is likely due to the buried sporulated oocysts of cats might be contaminating the soil and sand and these oocysts remain infectious for several months and can persist for over a year^49,71^.

#### Diagnostic performance of ELISA in detecting *Toxoplasma* antibodies in salivary samples

**Figure 1:**
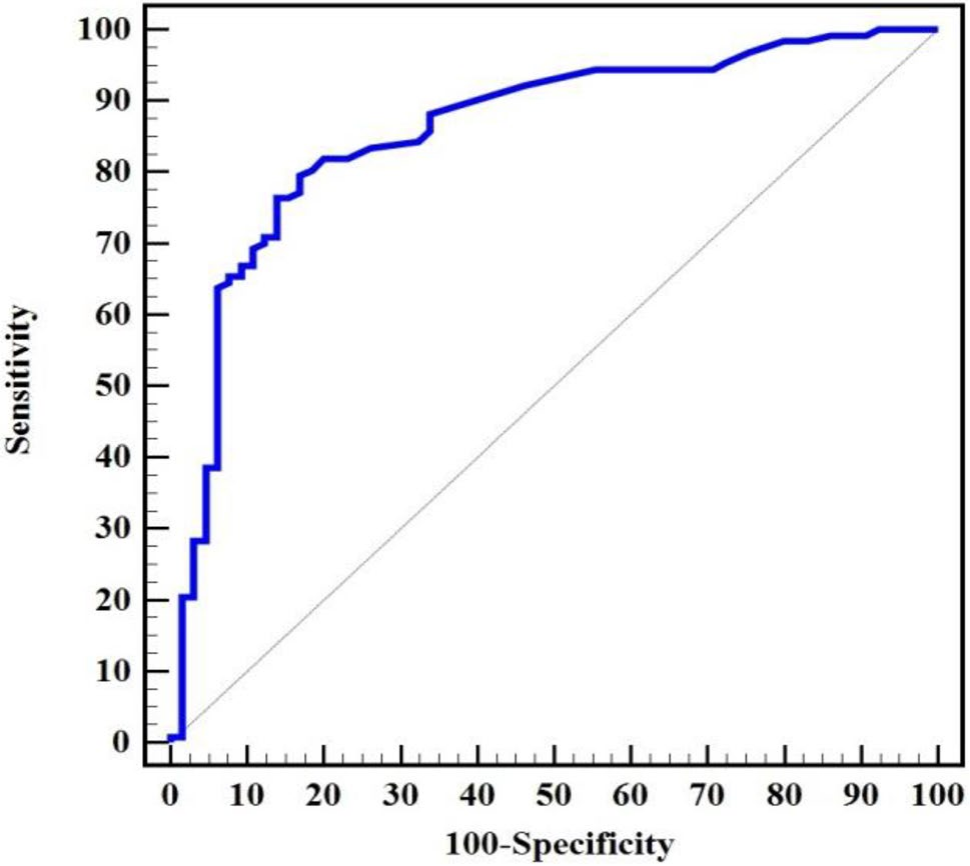
ROC curve for IgG saliva to discriminate positive IgG serum (n = 127) from negative IgG serum (n = 65).

Our findings revealed that 96 cases reported positive for IgG by the ELISA of salivary and serum samples. Conversely, 31 cases who were IgG negative in salivary samples were actually IgG seropositive, while 9 cases which were IgG negative when examining serum samples were found to be IgG positive when salivary samples were examined. 56 cases were IgG negative in both serum and salivary samples. Cohen’s Kappa coefficient (K) was 0.57 indicating fair agreement between serum and salivary ELISA techniques (Table 5). IgG detection demonstrated a sensitivity of 75.59% in salivary samples compared to serum samples, with a specificity of 86.15%. Additionally, PPV, NPV, and accuracy were 91.40%, 64.40%, and 79.17%, respectively, with an AUC of 0.809 (cut off > 2.969), (Table 5 and Fig. 1).

**Table 5:** Diagnostic performance of ELISA for detecting IgG in saliva compared to the detection of IgG in serum.

IgG	Serum				K (p)		Sensitivity	Specificity	PPV	NPV	Accuracy	AUC	95% C.I	Cut off^#^
	Negative (n = 65)		Positive (n = 127)											
	No	%	No	%										
Saliva														
Negative	56	86.2	31	24.4	0.570 (< 0.001*)		75.59	86.15	91.43	64.40	79.17	0.809	0.743–0.874	> 2.969
Positive	9	13.8	96	75.6										
χ^2^ (p)	66.146* (< 0.001*)													

χ^2^: Chi square test. K: Cohen’s Kappa coefficient.

*PPV* positive predictive value, *NPV* negative predictive value, *AUC* area under a curve, *CI* confidence intervals.

p: p value for association between different categories, *Statistically significant at p ≤ 0.05. ^#^Cut off was choose according to Youden index.

**Figure 2:**
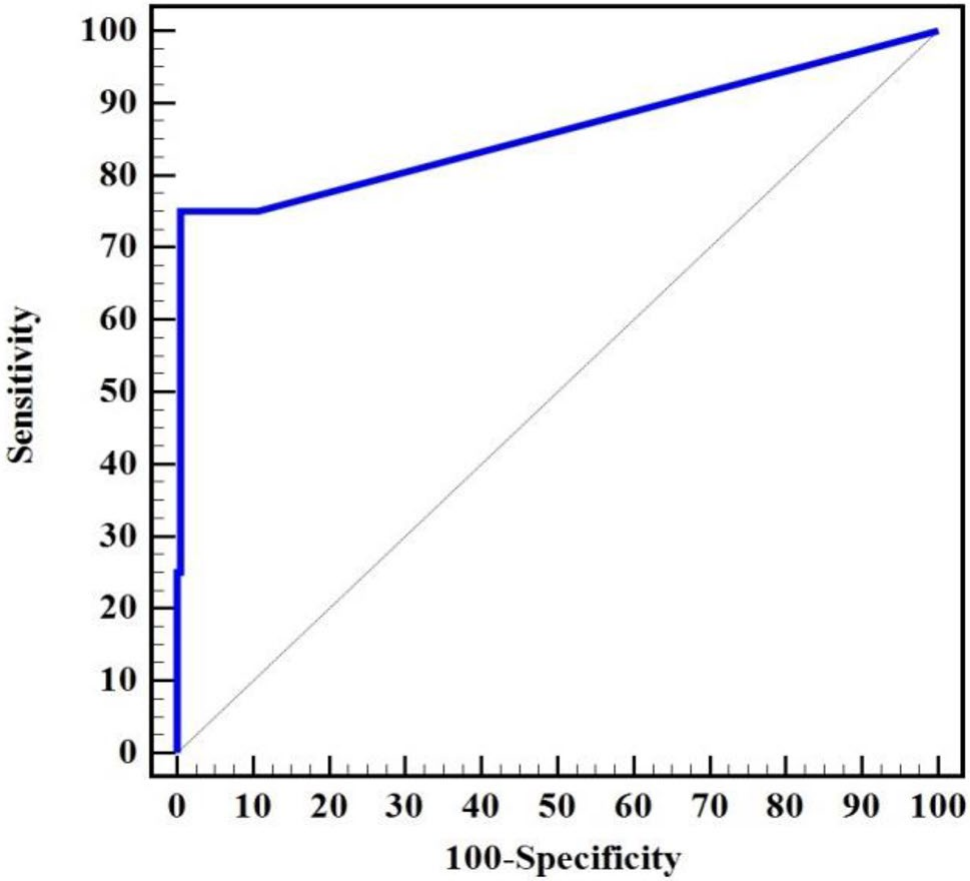
ROC curve for IgM saliva to discriminate positive IgM serum (n = 4) from negative IgM serum (n = 188).

In terms of ELISA detection of IgM, 3 cases tested positive while 187 cases tested negative in both serum and saliva samples. One sample showed positivity for IgM in saliva but negativity in serum and vice versa. Cohen’s Kappa coefficient (K) was 0.745 indicating excellent agreement between serum and salivary ELISA techniques. IgM in saliva exhibited a sensitivity of 75.0% and a remarkable specificity of 99.47% compared to IgM in serum. The PPV, NPV, accuracy, and AUC were 75.0%, 99.47%, 98.96%, and 0.859, respectively (Table 6, Fig. 2). In 2005, Singh et al.^53^, reported sensitivities for saliva IgG and IgM antibodies of 64% and 81.25%, respectively, with specificities of 94.67% and 85.71%, respectively, using cut-offs of 0.194 and 0.258, respectively. They concluded that saliva samples can serve as an alternative to serum samples for detecting anti- *Toxoplasma* immunoglobulins, particularly IgM, for diagnosing *Toxoplasma* encephalitis in HIV/AIDS patients. In 2019, Li et al.^10^, reported sensitivities, specificities, PPVs, and NPVs of *T. gondii* IgG and IgM tests on plasmonic gold chips in serum and saliva samples as follows: [(100% for IgG serum) (96.1%, 93.0%, 93.7%, and 95.7% respectively for IgG saliva) and (94.1%, 97.7%, 84.2%, and 99.2% respectively for IgM serum), and (100%, 95.4%, 73.9%, and 100% respectively for IgM saliva)], with successfully identified cut-offs of 0.3 and 1.5 for IgG and 0.35 and 0.32 for IgM in both serum and saliva samples respectively. Previous reports have also shown the presence of anti-*T. gondii* IgG, IgM, and IgA antibodies in saliva. Salivary IgA and IgG levels reflect those in serum, but they exhibit lower sensitivity and specificity values compared to the present study^17,72–74^.

**Table 6:** Diagnostic performance of ELISA for detecting IgM in saliva compared to the detection of IgM in serum.

IgM	Serum				K (p)		Sensitivity	Specificity	PPV	NPV	Accuracy	AUC	95% C.I.	Cut off^#^
	Negative (n = 188)		Positive (n = 4)											
	No	%	No	%										
Saliva														
Negative	187	99.5	1	25.0	0.745 (< 0.001*)		75.0	99.47	75.0	99.47	98.96	0.859	0.593–1.000	> 1.2
Positive	1	0.5	3	75.0										
χ^2^ (p)	106.474* (< 0.001*)													

χ^2^: Chi square test. K: Cohen’s Kappa coefficient.

*PPV* positive predictive value, *NPV* negative predictive value, *AUC* area under a curve, *CI* confidence intervals.

p: p value for association between different categories, *Statistically significant at p ≤ 0.05. ^#^Cut off was choose according to Youden index.

While many studies revealed the high levels of anti-*T. gondii* IgG, IgM and IgE along with pro-inflammatory cytokines such as IL-5, IL-6, and TNF-α in infected individuals, few have elucidated the mechanism underlying inflammation induction^75^. In silico study assist in examining the mechanism of PPI between *T. gondii* and pro-inflammatory cytokines, providing crucial insights into binding interface. Based on these findings, we screened numerous synthetic and natural therapies capable of inhibiting these PPI. In this aspect, bioinformatics analysis emerges as a promising tool for investigating the mechanism of *T. gondii* -induces inflammation, utilizing molecular docking techniques and exploring novel target therapies through drug docking. Further study can furnish additional data about the mechanisms underlying *T. gondii* -induced inflammation and severe illness, while also expanding the scope for explore new target therapies.

### Molecular docking

**Figure 3:**
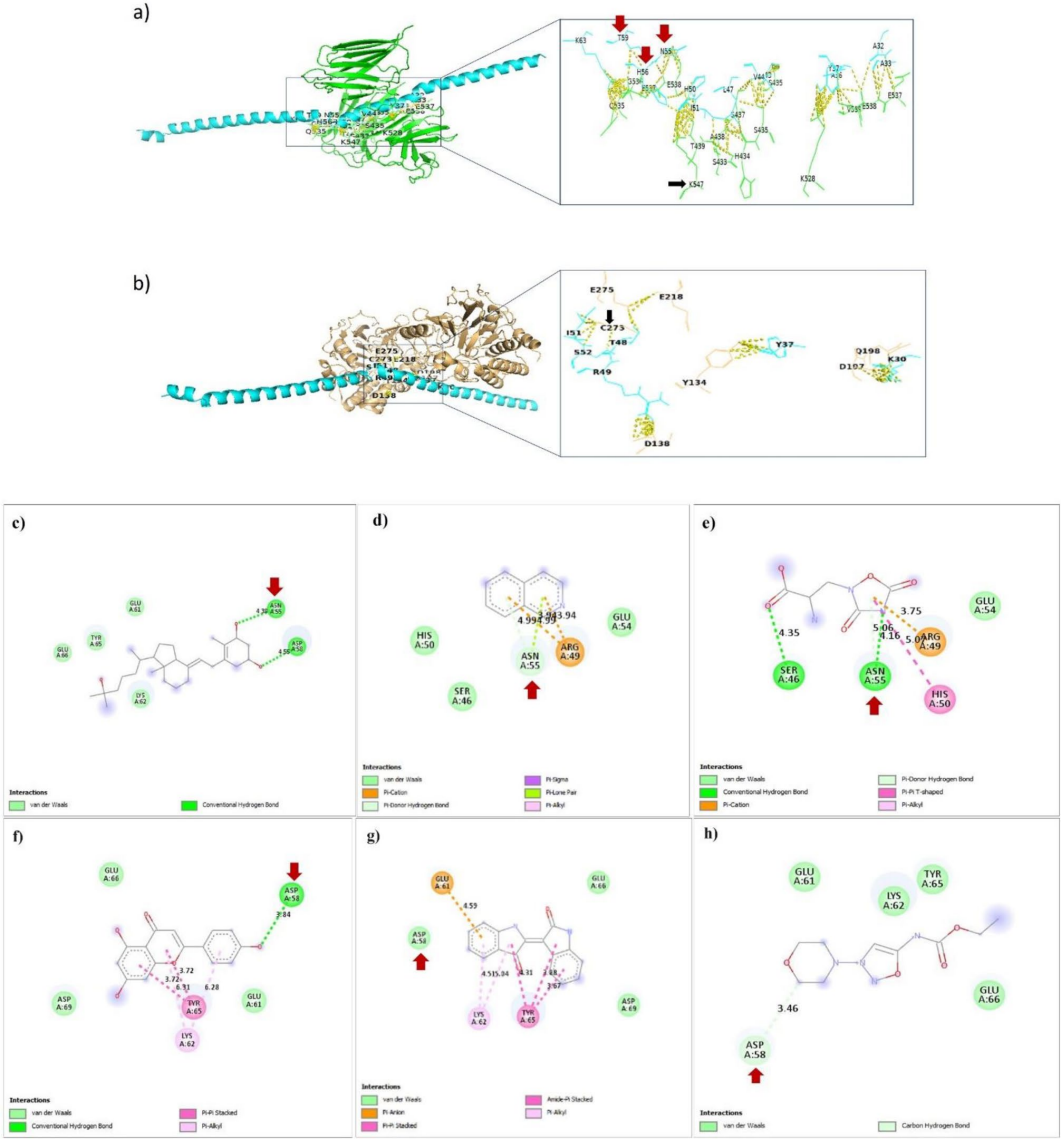
Upper panel showed the molecular docking of TgERP to (**a)** Lamin B1 and (**b**) Ki67. Down panel showed the drug docking of (**c**) Calitriol, (**d**) Isoquinolone, (**e**) Quasqualic, (**f**) Apigenin, (**g**) Indirubin, (**h**) Molsidomine to TgERB.

We did not observe any interaction between TgERP and pro-inflammatory interleukins including IL-6, IL-17, or anti-inflammatory interleukin IL-10 with a distance cut-off 3.5Å or 4Å. This data suggests that TgERP might enhance cellular dysfunction through interactions with proteins other than interleukins. As shown in Fig. 3, there are PPI between A29 K30 Y37 T48 R49 I51 S52 of TgERP and residue E218 of chain B, as well as residues Y134, D138, D197, Q198, **C273**, and E275 of chain B Ki67 with distance cut-off of 3.5Å. Notably, C273 is identified as a critical residue of Ki67. Additionally, we observed PPI between A29 A33 A36 Y37 G43 V44 L47 H50 I51 N55 H56 T59 K63 of TgERP and S433 H434 S435 K528 E537 E538 V539 K547 of chain B as well as S435 S437 A438 T439 Q535 G536 E537 E538 of chain C of LB1 with a cut-off 3.5Å.

### Drug docking

**Figure 4:**
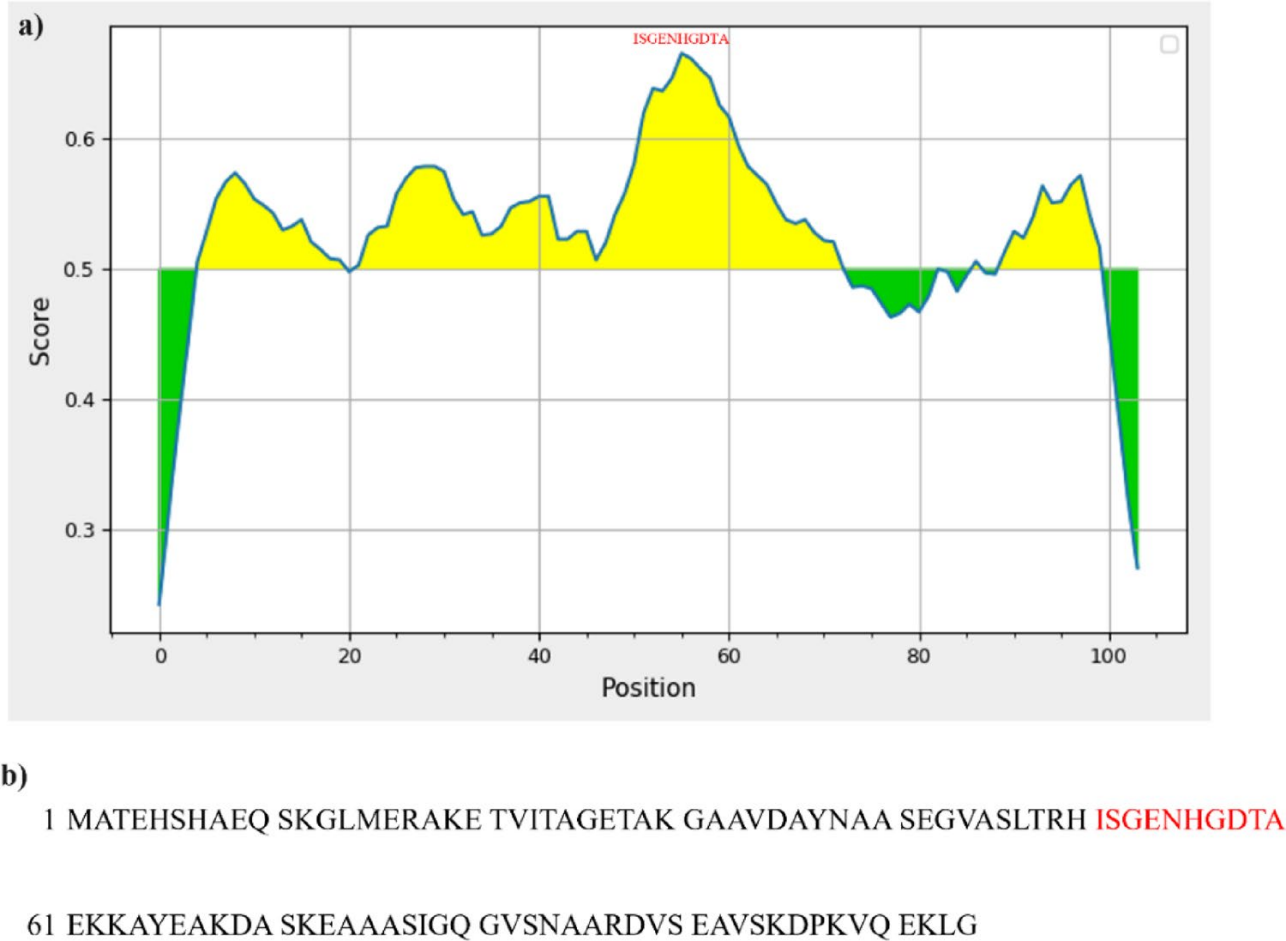
(**a**) Histogram plot showing the score of B-cell epitopes of TgERP using BepiPred-2.0 epitope predictor in IEDB-analysis database, (**b**) Sequence of TgERP as published by Hill D et al.^25^^,^^83^.

As shown in Fig. 4, utilizing the immune epitope database to identify the epitope of TgERP showed that the sequence extending from S52 to D58 showed a high score. Table 7 demonstrates that aminopterin exhibits the highest affinity towards TgERB then calcitriol, kampferol, apigenin, quercetin, molsidomine, quisqualic, isoquinolone, coumarin and thymoquinone in descending order. Studying the binding interface between the screened therapies and TgERB offers novel mechanisms for the treatment of toxoplasmosis with either synthetic therapies or natural products. Calcitriol docked TgERP by binding to Asn55 and Asp58 via hydrogen bonds (HBs) with distances 4.38 Å and 4.55 Å, respectively. isoquinolone and quasquialic docked TgERP via binding to Asn55 with two Pi-donor HB at equal distances of 4.99Å. Apigenin docks TgERP by binding to Asp58 via HB with a distance of 3.84 Å. Additionally, Indirubin dockes to TgERP by binding to Asp58 (VDW). In addition, Molsidomine, an approved therapy as a vasodilator^76^, dockes to TgERP by binding to Asp58 with carbon HB at a distance of 3.46 Å. Although aminopterin docked TgERP with the lowest binding energy − 92.74 kcal/Mol in addition to target Asp58 by two HB at distances 3.73 Å, 4.15 Å, its production was discontinued due to its adverse effects such as hepatotoxicity^77^.

**Table 7:** Drug docking of synthetic and natural therapies to TgERP.

Drug docking				
Name	Energy Kcal/Mol	VDW	Hbond	Elec
Aminopterin	− 92.74	− 70.89	− 22.53	0.68
Calcitriol	− 75.5	− 70.59	− 4.91	0
Kaemferol	− 71.69	− 67.59	− 4.1	0
Apigenin	− 69.16	− 66.66	− 2.5	0
Indirubin	− 94.94	− 64.94	0	0
Quercetin	− 63.79	− 61.29	− 2.5	0
Molsidomine	− 62.862	− 55.98	− 6.87	0
Quisqualic	− 55.64	− 33.2	− 20.98	− 1.46
Isoquinolone	− 44.42	− 37.42	− 7	0
Coumarin	− 43.39	− 39.15	− 4.24	0
Thymoquinone	− 42.61	− 40.11	− 2.5	0

From the previous, it is evident that natural medicinal products such as calcitriol, isoquinolone, quisqualic, and apigenin indirubin, exhibit pronounced benefits when targeting the same critical residues including N55 H56 D58 with high affinity. Particularly, analysis of data collected from the B-cell epitope predictor revealed that the peptide 52-SGENHGDTAE-61 represents the most probable B-cell epitope with scores ranging from 0.617 to 0.666. These findings aid in understanding the mechanism of TgERP induces inflammatory effects by enhancing antibody binding to the B-cell epitope, subsequently, TgERP can elicit the cytokines storm in infected patients. Additionally, our study demonstrates that the screened drugs efficiently target the B-cell epitope rather than T-cell ones, thereby enabling them to counteract the inflammatory action of TgERP-induced cytokines storm without impairing the immune response of T-cell. Besides numerous studies have evaluated the efficacy of the screened therapies to antagonize inflammation across various diseases. In this aspect, calcitriol, an active metabolite of vitamin D, acts as a regulator of calcium levels and the immune system^78^. The antioxidant and anti-inflammatory properties of apigenin and quercetin extracted from Matricaria recutita, have been established in relief of gastrointestinal disorders^79,80^. Indirubin, derived from plants such as *Isatis tinctoria*, *Polygonum tinctorium*, and *Strobilanthes cusia*, has been shown clinical relevance in the treatment of chronic myelocytic leukemia according as reported by Hu in 2015^81^.

Data collected from molecular docking revealed that interaction between His50, Ile51 of TgERP, and Lys547 of LB may hinder the action of LB where Lys547 is a target residue for SUMOylation that is one of the post-translational modifications regulating nuclear cellular processes by altering protein localization and function^82^. Our findings propose a novel mechanism for *T. gondii*-induced cell lysis, oxidative stress, and inflammation through the disruption of LB, a regulator of nuclear envelope integrity resulting in chromatin externalization, a hallmark of NETs^23^.

Ki67, a nuclear transcription factor, plays an essential role in the dephosphorylation of phosphor-serine (pSer) and phosphor-threonine (pThr) of many proteins involved in numerous biological processes such as glycogen metabolism, cell cycle regulation, smooth muscle contraction, and protein synthesis. Ki67 serves as a prognostic and diagnostic tool for cell proliferation. It has been demonstrated that the C terminus of Ki67 interacts with heterochromatin protein 1 family such as PP1. Our study showed that TgERP interacts with Ki67 by binding to many amino residues especially C273 which is a critical residue for its integrity. Consistently, it has been demonstrated that microcystin toxin inhibits KI67 via binding to Cys273^83^. Additionally, He JJ et al.^84^ suggested another mechanism of *T. gondii-*induced carcinogenesis via impairing the lipid synthesis and the immune response. Our study suggests another potential mechanism of *T. gondii*-induced carcinogenesis through the interaction between TgERP and Ki67 resulting in disturbances in chromatin organization, cell division, and cell integrity, ultimately leading to the transformation of normal cells into malignant ones. In line with this, Shen et al.^85^, revealed an association between *T. gondii* infection and B-cell lymphoma. It was revealed that microorganisms produce various PP1 inhibitors such as microcystin and okadaic acid^86^. Dysregulation of PP1 activity has been associated with numerous diseases, including cancer and heart failure^87^. Our finding suggests that TgERP may disrupt many vital biological processes by interfering with the complex structure of Ki67/PP1. Subsequently, TgERP may induce necroptosis, an unprogrammed cell death, in the host cell. It is consistent findings showing that inhibiting Ki67 correlates with overexpression of RIPK3 in various inflammatory diseases and cancers^88^.

Analysis of the drug docking data revealed that Asp58 Lys62 Tyr65 Glu61 in TgERP are common target residues for aminopterin, apigenin, indirubin, molsidomine, and calcitriol. Additionally, Glu61 and Glu66 of TgERP were identified as common target residues for aminopterin, apigenin, indirubin, molsidomine, calcitriol, and quercetin, however the former is also serves as a target for quinapril (Supplementary Information).

Of note, Asn55 serves as a unique target residue for isoquinolone, quisqualicm, and calcitriol. Furthermore, quisqualic and isoquinolone bind to the same residues of TgERP including Ser46 Asn55 Arg49 His50. The former exhibited a lower binding energy compared to the latter. The current study reveals a novel mechanism of action for the screened phytotherapies isoquinolone, quisqualic, and calcitriol in preventing the binding of TgERP with Ki67. Additionally, TgERP interacts with LB by binding with multiple residues, especially C273, which is essential for maintaining the integrity of the LB structure. Also, it has been demonstrated that *T. gondii*-induced NET, a cellular lysis process, is triggered via several mechanisms, including the disassembly of LB into intact full‐length molecules, the production of reactive oxygen species, the activation of protein kinase C (PKC), extracellular‐signal‐regulated kinase (ERK/MAPK), and phosphatidylinositide 3 kinase (PI3K)/Akt^89^.

## Conclusion

Collectively, our data showed that ELISA examination of saliva to detect anti-*T. gondii* immunoglobulins can effectively distinguish between positive and negative samples, making it as a promising diagnostic approach. Furthermore, the synthetic drugs and natural products examined in our study target specific amino acids residues of TgERP located at the same binding interface as LB1 and Ki67, thereby impeding their interaction. These findings offer potential strategies to mitigate the inflammatory effects and consequences of toxoplasmosis. Incorporating the examined drugs to treatment regimen for toxoplasmosis may help in contracting its inflammatory consequences. 

### Supplementary Information

Supplementary Information.

## Data Availability

All data generated or analyzed during this study are included in this article.

## Abbreviations

AUC: Area under the curve
K: Cohen’s Kappa coefficient
ELISA: Enzyme-linked immunosorbent assay
*H. pylori*: *Helicobacter pylori*
H.B.: Hydrogen bond
Ig: Immunoglobulin
LB: Lamin B
IL: Interleukin
LB1: Lamin B1
NPV: Negative predictive values
NET: Neutrophil extracellular trap
PPV: Positive predictive values
ROC: Receiver operating characteristic curve
T. gondii: *Toxoplasma gondii*
TgERP: *T. gondii* Embryogenesis-related protein
