# Supplementary figures and images for: In silico study to explore the mechanism of Toxoplasma-induced inflammation and target therapy based on sero and salivary Toxoplasma
Source: Sci Rep. 2024 Jun 13;14:13600. doi: 10.1038/s41598-024-63735-z (PMC11169245; doi:10.1038/s41598-024-63735-z)

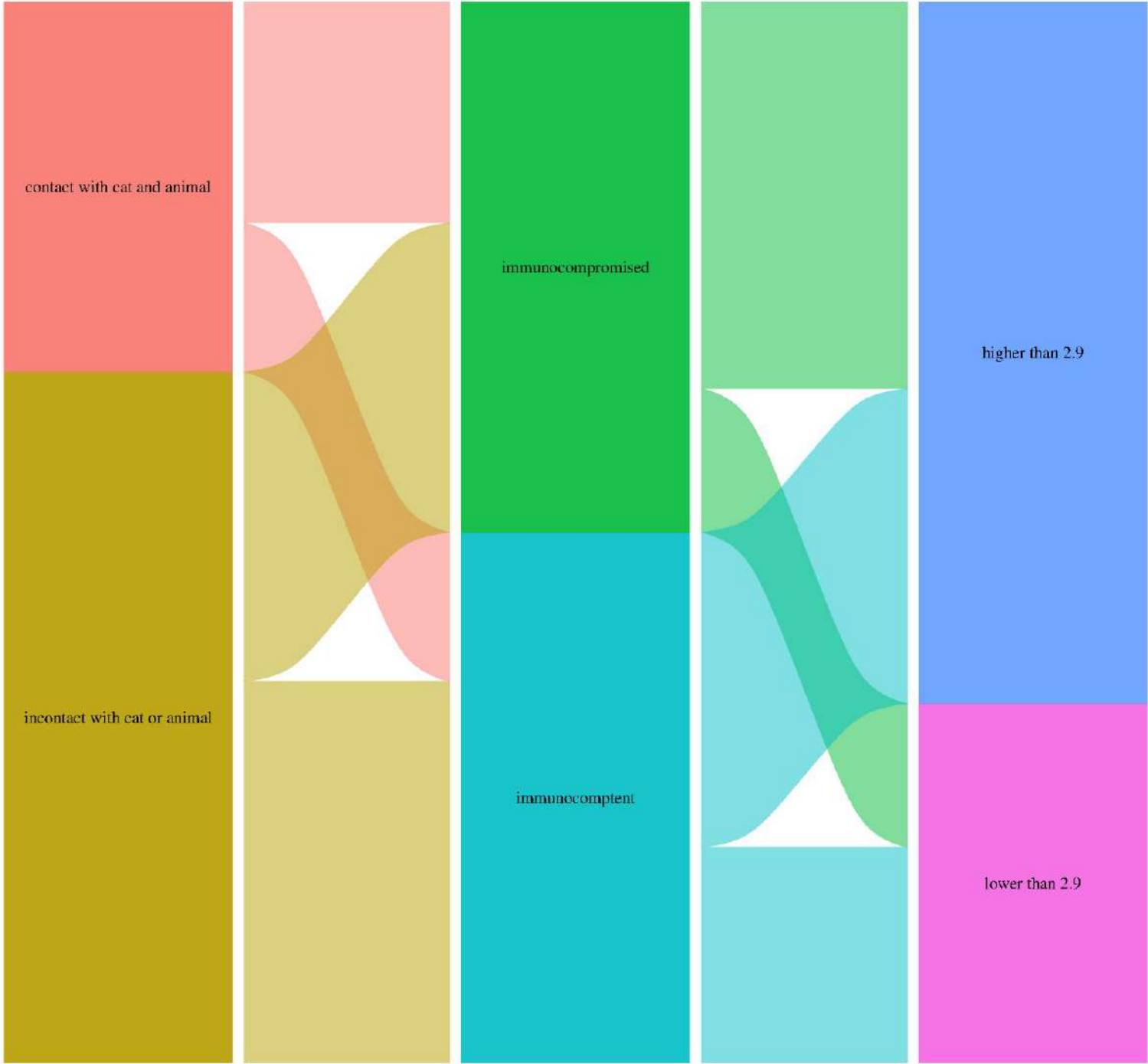

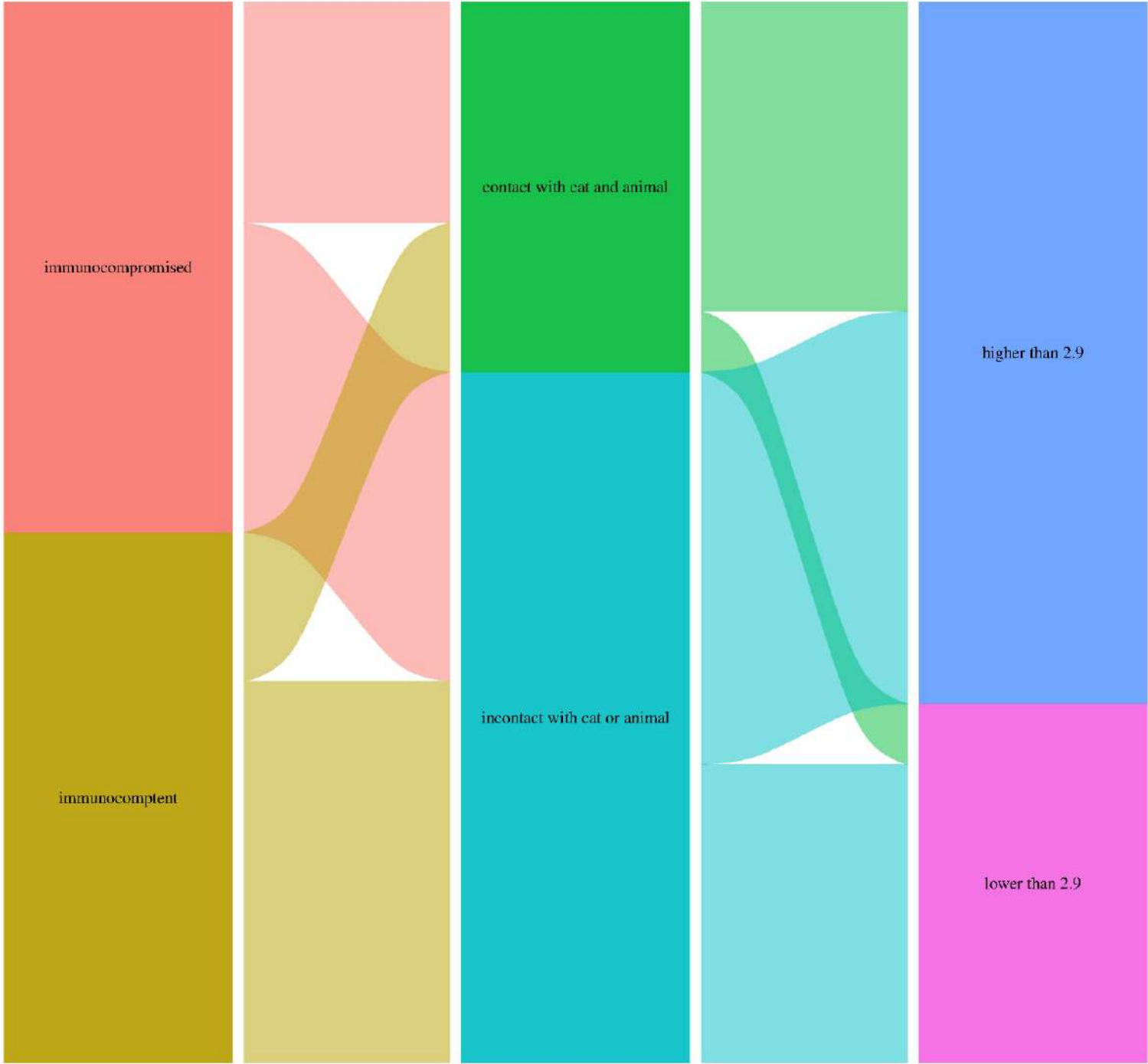

contact with cat and animal

incontact with cat or animal

higher than 2.9

lower than 2.9

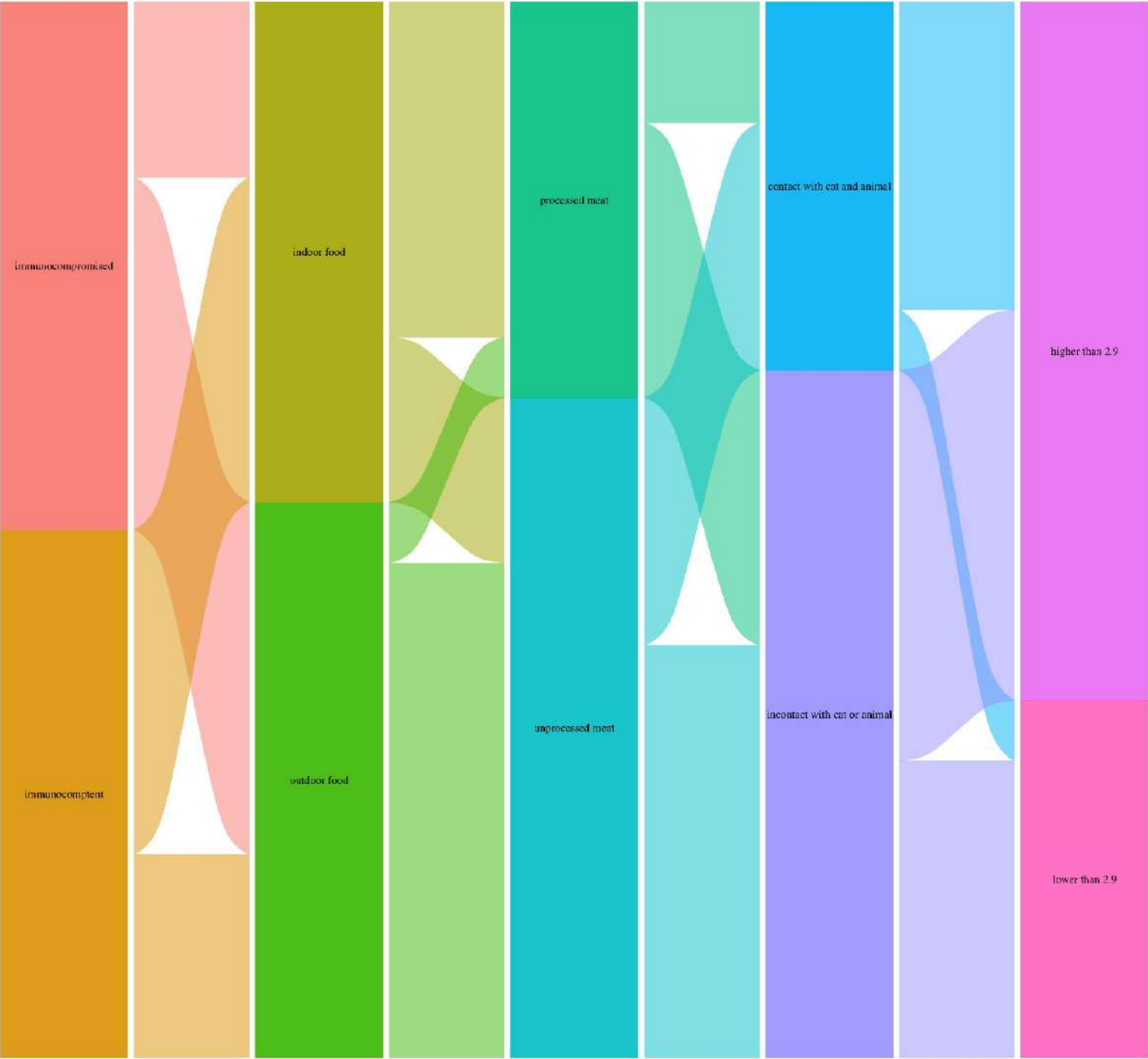

Supplement: Supplementary file 1 — Supplementary Information. [file 41598_2024_63735_MOESM1_ESM.pdf]
